# Nitrogen and Phosphorus Co-doped Porous Carbon for High-Performance Supercapacitors

**DOI:** 10.3389/fchem.2020.00105

**Published:** 2020-02-20

**Authors:** Jiaming Zhou, Shewen Ye, Qinqin Zeng, Hui Yang, Jiahao Chen, Ziting Guo, Honghui Jiang, Karthikeyan Rajan

**Affiliations:** School of Materials Science and Engineering, Jiangxi University of Science and Technology, Ganzhou, China

**Keywords:** biomass, porous carbon, supercapacitor, *Eleocharis dulcis*, N/P co-doped

## Abstract

As one of the most promising fast energy storage devices, supercapacitor has been attracting intense attention for many emerging applications. However, how to enhance the electrochemical performance of electrode materials is still the main issue among various researches. In this paper, hierarchical porous carbons derived from *Eleocharis dulcis* has been prepared by chemical activation process with the aid of KOH at elevated temperature. Results show that the N, P co-doped porous carbon exhibits excellent electrochemical performance, it owns a specific capacitance of 340.2 F/g at 1 A/g, and obtains outstanding cycling stability of 96.9% of capacitance retention at 10 A/g after 5,000 cycles in a three-electrode system. Moreover, in the two-electrode system, the product still maintains a high specific capacitance of 227.2 F/g at 1 A/g, and achieves good electrochemical cycle stability (94.2% of capacitance retention at 10 A/g after 10,000 cycles); besides, its power/energy density are 3694.084 and 26.289 Wh/kg, respectively. Therefore, the combination of facile synthesis strategy and excellent electrochemical performance makes *Eleocharis dulcis*-based porous carbon as a promising electrode material for supercapacitor.

## Introduction

Rapid development of global economy, the depletion of chemical fuels and the ever-worsening environment are intensified with the continuous growth of the population, which increases the demand for clean sustainable energy. Thence it requires the development of efficient and clean energy storage devices (Wang et al., 2016; Liu et al., 2017b; Yang et al., 2019). Among them, the traditional Lithium-ion batteries will generate quantum and form lithium dendrites under high-power operation, the supercapacitors have distinctive properties such as excellent power density, rapid charging and discharging speed and superior cycle stability, is considered the best substitute for lithium-ion batteries (Zhao et al., 2013; Shao et al., 2018; Li et al., 2019). Although supercapacitors exhibit excellent properties, low specific capacity and energy density (typically <10 Wh/kg) toward large scale commercial devices are still major constraints (Winter and Brodd, 2004; Liu et al., 2017b).

Electrode materials are the important constituent which affect the properties of supercapacitor. Traditionally, different allotropes of carbon materials are used as an electrode in energy storage applications. Among them, sustainable biomass derived carbons are individual class of materials, with the advantage of low-cost, abundant and sustainable in nature, excellent electrical conductivity and specific surface area (SSA) (Pandolfo and Hollenkamp, 2006; Jiang et al., 2013; Titirici et al., 2015; Gong et al., 2017; He et al., 2018). Number of researchers have derived carbon from different biomass sources such as *Perilla frutescens* (Liu et al., 2017a), Rice straw (Liu et al., 2018), Peanut shells (Xiao et al., 2018), Buckwheat flour (Huang et al., 2019), Peach gum (Lin et al., 2019), and Bamboo (Zhang et al., 2018). By using KOH through chemical activation Cheng et al. (2016) have prepared flexible carbon fiber aerogel from natural cotton and achieved specific capacitance of 283 F/g at 1 A/g. Similarly, the graded porous carbon material derived from walnut shells resulted the capacitance of 462 F/g at 1 A/g (Wang et al., 2019). Besides, the specific surface area of carbon materials derived from seaweed microspheres show as high as 1337.9 m^2^/g, the capacitance led to 309 F/g at 1 A/g, with the capacitance retention rate of 92% at 20 A/g with 10,000 cycles (Zhu et al., 2018). Therefore, it is necessary to reveal the relationship between different biomass sources and their relation to specific surface area and specific capacitance.

Due to their limited number of active sites on microporous carbon, it is important to investigate the improvement of electrical conductivity and electronegativity properties. In this work we have fabricated the nitrogen and phosphorus co-doped microporous derived carbon for supercapacitor application. The reason why biomass derived carbon materials can show excellent capacitance performance, is closely related to the incorporation of trace elements such as nitrogen and phosphorus into carbon materials in the carbonization process (Shen and Fan, 2013; Chen et al., 2014; Zhao et al., 2017). The presence of nitrogen in carbon materials is expected to improve the electron conductivity of the materials (Chen et al., 2013). Phosphorus contained materials could help to improve the electronegativity of carbon through the combination of lone pair electron nitrogen and carbon, thereby enhance the hydrophilicity of carbon materials. The nitrogen-containing groups on the surface are alkaline, which is conducive to ion adsorption (Shen and Fan, 2013). Nitrogen and phosphorus are belong to the same group in periodic table, however phosphorous possess higher electron-donor capacity, which is useful to achieve stable capacitive property (Zhao et al., 2017). Therefore, we believe that presence of nitrogen and phosphorus in carbon would greatly improve the capacitive performance.

China cultivates the largest quantities of *Eleocharis dulcis* (ED) in the world with the annual output of up to 1.75 million tons. *Eleocharis dulcis* is rich in trace elements such as nitrogen and phosphorous, and the phosphorus element is higher in root vegetables (Bao et al., 2018). Therefore, deriving carbon from *Eleocharis dulcis* without affecting the existing N and P elements could help to enhance the specific capacitance (Panja et al., 2015). In this work, we have derived the microporous activated carbon material from ED by chemical activation process and carbonized. To the results of N_2_ adsorption-desorption isothermal analysis describes the highest, specific surface area of 2,454 m^2^/g with the specific capacitance of 340.2 F/g at 1 A/g.

## Experimental

### Materials Synthesis

In general, ED were freeze-dried and grinded, after screening through a 70-mesh sieve, the obtained powders were mixed with certain amount of KOH solution, then dried at 80°C for 12 h. The as-prepared precursors were transferred into a tube furnace, after that, the powders were treated at an elevated temperature (800°C for 1 h) under N_2_ atmosphere. The obtained carbon material was washed by 2 M HNO_3_ to remove the impurities, subsequently, the final products were washed with deionized water several times, then dried at 60°C for 24 h. The samples treated with different KOH/ED mass ratio (1:1, 2:1, 3:1) were referred to referred to as NPC-1, NPC-2, and NPC-3, respectively.

### Materials Characterization

Scanning electron microscope (SEM, ZEISS Sigma) and transmission electron microscope (TEM, JEOL, JEM-2010, Japan) are used to analyze the morphology and microstructure of the samples. The phase and results of the samples are analyzed by X-ray diffraction (XRD, Empyrean). Raman spectroscopy (excitation beam wavelength 532 nm) is used to analyze the graphitization degree of materials. The nitrogen adsorption and desorption isotherms and the specific surface area, pore diameter distribution and pore volume of the samples are measured by N_2_ adsorption-desorption experiment (Micromeritics, ASAP 2010M, USA).

### Electrochemical Measurements

All electrochemical measurements were performed at CHI760E electrochemical workstation (Chen Hua Shanghai). The electrochemical test used a standard three-electrode system, in which Hg/HgO is used as the reference electrode, Pt electrode was used as the counter electrode, the prepared NPCs were used as the working electrodes, and 6 M KOH aqueous solution as the electrolyte. The working electrodes were prepared according to the following procedure: 80 wt.% NPCs, 10 wt.% acetylene black, and 10 wt.% PVDF (binder) were thoroughly mixed in the N-methyl-2-pyrrolidone (NMP) solvent to obtain a uniform semi-fluid slurry; the prepared slurry was coated onto carbon cloth, and then dried in a vacuum oven at 60°C for 12 h. The coating mass of active material in each working electrode is about 2 mg/cm^2^. The cyclic voltammetry and galvanostatic charge/discharge (GCD) curve had been performed at various scanning rate/current density, and the corresponding electrochemical impedance spectroscopy (EIS) was tested at an open circuit voltage (frequency range: 0.01–100 kHz, amplitude: 5 mV). The calculation details were provided in Supplementary Material.

## Results and Discussion

**Figure 1:**
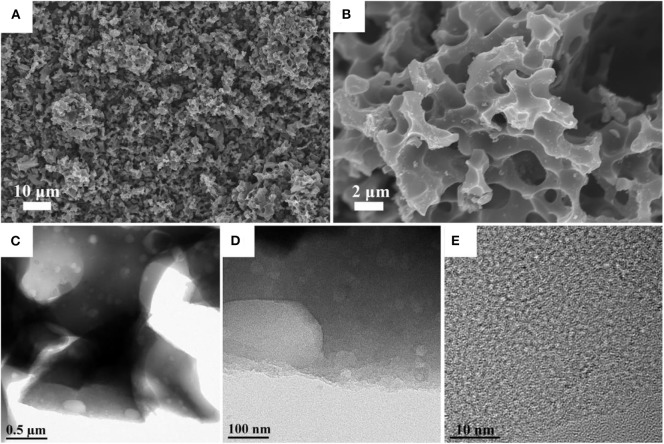
**(A,B)** SEM images of the NPC-3. **(C,D)** TEM images of the NPC-3 under different magnifications. **(E)** HRTEM image of the NPC-3.

The scanning electron microscopy (SEM) analysis was used to analyze the morphological significance of synthesized carbon materials. Figure 1 shows the low and high magnification images of NPC-3. The microstructure images reveal the presence of large hierarchical porous on the derived carbon The diameter of these large pore structures varies from few to several micrometers, Compared with the NPC-1 (Figures S2A,B) and NPC-2 (Figures S2C,D), the NPC-3 has more uniform hole distribution with macropores structure. The structure of NPC-3 was further studied by TEM, according to the TEM results (Figures 1C–E), there are many randomly distributed mesopores, which connect with the macropores and form a hierarchical porous structure. It is well-stated that KOH activation leads to the formation of abundant micropores due to its corrosive nature (Bleda-Martínez et al., 2005; Guan et al., 2009), the reaction mechanism is described as follows:

(1) $$\begin{array}{l} \text{KOH}+\text{C}↔\text{K}+\text{C}{\text{O}}_{2}+{\text{H}}_{2}\text{O} \end{array}$$

**Figure 2:**
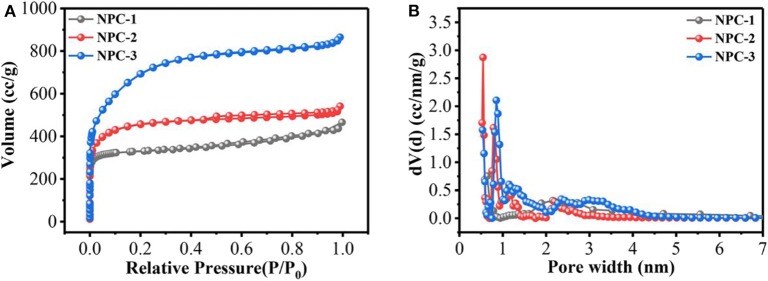
**(A)** Nitrogen adsorption/desorption isotherms. **(B)** Pore size distribution curve of NPC.

The electrochemical performance of carbon-based materials are greatly depends upon its solid/electrolyte interface, and its porous structure (Salanne et al., 2016). The contribution of micropores and mesopores to the specific capacitance were not discussed in detail. Previous reports indicated that neither micropores nor mesopores influenced the energy/power density (Kim et al., 2013). Besides, Lei et al. found that hierarchical micropores with wider size distribution led to high energy storage, which provided a fast transportation pathway for ions (Lei et al., 2011). The nitrogen adsorption-desorption analysis was conducted for the synthesized carbon and the results are shown in Table 1. Figures 2A,B represents the N_2_ adsorption-desorption isothermal and pore size distributions of NPC-1, NPC-2, and NPC-3. Results reveal that all samples are composed of type I and type IV isotherms. The sharp adsorption of N_2_ at low relative pressure (0~0.1) indicates the presence of micropores in the porous carbon structure. The hysteresis loops at higher relative pressure represents the presence of mesopores structure in NPC-3; the curve shape near high relative pressure region indicates there are small amount of macropores structure (Lv et al., 2012). The pore size distribution of the samples has been tested. As can be seen from Table 1, with the increase of KOH, the BET surface area (S_BET_) and total pore volumes (V_total_) of samples increased from 1,063 to 2,454 m^2^/g and from 0.352 to 1.345 cm^3^/g, respectively. The electrode material with high specific surface area can provide abundant electrochemical active sites and enhance the effective charge storage area between electrode and electrolyte, thus improving the performance of supercapacitor (Xu et al., 2018).

**Table 1:** Adsorption parameters of different samples calculated from N_2_ adsorption isotherms.

**Samples**	**S _**BET**_ (m^**2**^/g)**	**S *_**micro**_* (m^**2**^/g)**	**V *_**micro**_* (cm^**3**^ /g)**	**V _**total**_ (cm^**3**^ /g)**	**D _**Average**_ (nm)**
NPC-1	1,063	889	0.302	0.352	0.415
NPC-2	1,708	1,455	0.596	0.729	0.545
NPC-3	2,454	1,522	0.650	1.345	0.852

**Figure 3:**
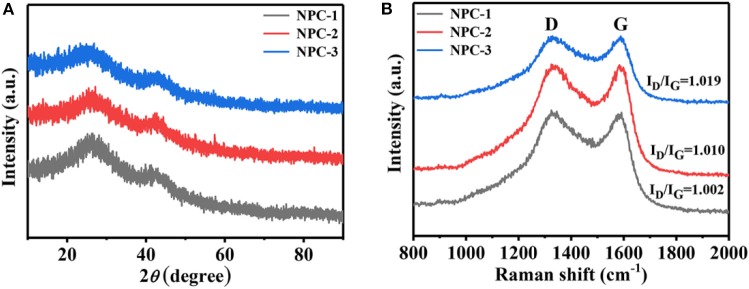
**(A)** XRD patterns. **(B)** Raman spectrum.

Figure 3A shows the X-ray diffraction (XRD) pattern and Raman spectra (Figure 3B) of synthesized carbon samples derived from ED. The broad characteristic peaks at 25.9 and 43.1°C are corresponding to the (002) and (100) crystal planes of carbon materials, respectively (Wan et al., 2015). Increasing the mass ratio of KOH to ED leads to the broadening of diffraction peak (002) and (100). This indicates that the KOH can significantly affect the orderings of the crystal planes. Raman spectroscopy of Figure 3B describes the D-band (at 1,333 cm^−1^) G-band (at 1,589 cm^−1^). The D-band is attributed to disordered nature of graphitic planes and G-band ascribed to ordered planes due to sp^2^ hybrid carbon stretching vibration. More importantly, I_*D*_/I_*G*_ reflects the degree of graphitization of the material (Ferrari et al., 2006; Zhou et al., 2014), and the I_*D*_/I_*G*_ of NPC-1, NPC-2, and NPC-3 are 1.019, 1.010, and 1.002, respectively. The result indicates that the higher KOH ratio inhibits the graphitization of the material, raises the disorder of the microstructure of the material. This is consistent with the XRD results.

**Figure 4:**
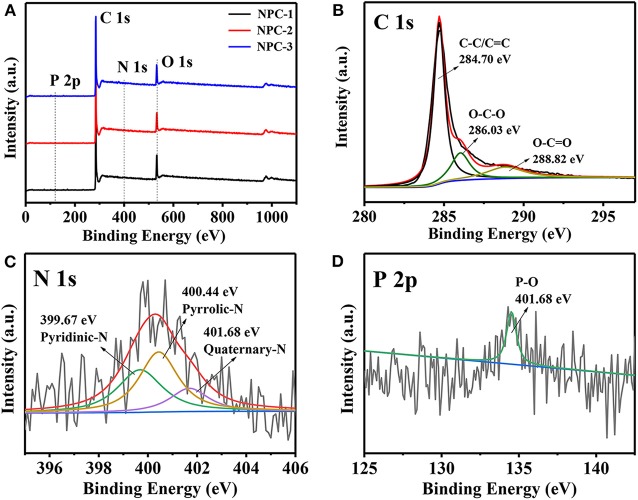
**(A)** XPS survey spectra of NPCs. **(B)** C1s **(C)** N1s and **(D)** P2p XPS spectra of the NPC-3.

The surface chemical properties of NPCs are investigated by X-ray photoelectron spectroscopy (XPS) measurements. The characteristic peak for C1s (~284.60 eV), N1s (~400.45 eV), O1s (~532.64 eV), and P2p (~134.40 eV) were observed in the spectrum (Figure 4A). The C1s spectra (Figure 4B) of the NPC-3 display three distinct characteristic peaks at 284.70, 286.03, and 288.82 eV, they are corresponding to different carbon functional groups of C-C or C=C, O-C-O, and O-C=O, respectively (Li et al., 2016). N1s spectra contains three peaks located at 399.67, 400.44, and 401.68 eV, corresponding to pyridinic-N, pyrrolic-N, and quaternary-N (Figure 4C). Pyridinic-N and pyrrolic-N species have positive charge, they can enhance the electron transfer at high current density, while quaternary-nitrogen can increase the conductivity of materials (Yang et al., 2019). In addition, Figure 4D shows a P2p spectrum with a peak value of 134.53 eV, representing P-O functional group. According to Table 2, the doping amount of P is about 0.18~0.25%. P has a higher electron delivery capacity than N, which can significantly improve the charge storage and transport capacity of carbon materials. Therefore, N and P doping are beneficial to the electrochemical performance of supercapacitors. As we all know, the N content of NPCs decreases with the increase of KOH mass, while it won't affect the P content. Interested, O at% is negatively correlated with P at%, this should be attributed to the part of P atoms, which are directly bonded to C atoms, and do not bind the edges of the carbon lattice by P-O.

**Table 2:** The contents of C, N, P and O in NPCs from XPS analysis.

**Samples**	**C (at%)**	**N (at%)**	**O (at%)**	**P (at%)**
NPC-1	87.43	1.24	11.08	0.25
NPC-2	87.96	1.07	10.68	0.29
NPC-3	85.98	0.95	12.89	0.18

**Figure 5:**
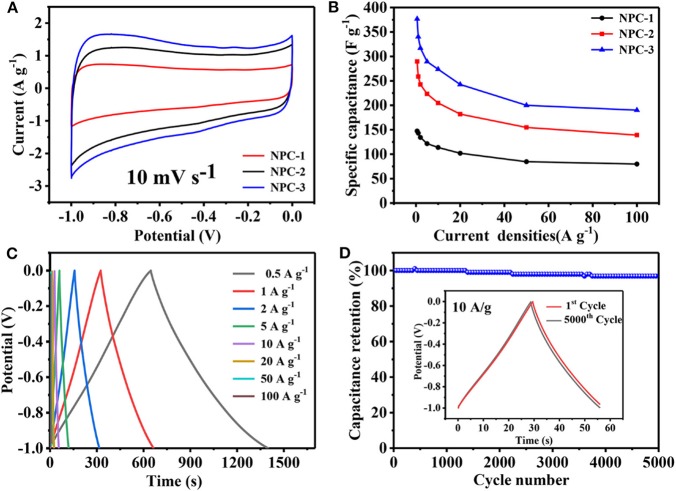
**(A)** CV curves of PCs at10 mV/s. **(B)** GCD curves of NPC-3 at different current density. **(C)** Variation of specific capacitances vs. the current density of NPCs. **(D)** Cycling performance of NPC-3 at 10 A/g.

The electrochemical characterizations were carried out for the synthesized carbon materials as shown in Figure 5. All the samples are tested under 6 M KOH electrolyte through the three-electrode system. As shown in Figure 5A, all the CV curves of NPCs represent the quasi rectangular shape at 10 mV/s sweeping potential. This indicates that the charge can be reassembled quickly when the voltage is turned, it reveals the material has good rate capability and cycle performance (Xu et al., 2019). It can be seen that the CV curve of NPC-3 has the largest area which depicts the highest specific capacitance of, which represents 340.2 F/g at 1 A/g. Figure 5C shows the galvanostatic charge and discharge (GCD) curves of NPC-3 under different current densities. All curves show symmetrical triangular shape without any voltage drop, indicating that the material has good rate capability and cycle performance. However, the GCD curve is not strictly symmetric due to pseudocapacitive effect caused by the presence of N and P. Figure 5B plotted against capacitance with respect to different current densities. All the NPCs signify the decrease in its specific capacitance with respect to the increase in current density. Besides, the cycling stability of the NPC-3 has been resulted to 96.9% of the initial specific capacitance after 5,000 cycles at 10 A/g, this could be due to the 3D structure of carbon materials and the contribution of phosphorus and nitrogen functional groups, NPC-3 has good cycle stability.

In summary we summarize the reasons for the superior electrochemical performance of NPC-3: (1) The co-doped of N and P atoms produces more active sites, which leads to the increase of conductivity and electronegativity of porous carbon, increases the hydrophilicity of the material, and then increases the effective specific surface area, leading to the common effects of capacitance and pseudo capacitance (Chen et al., 2015). (2) The micropores in the hierarchical porous carbon can be used to store charge, and mesoporous and macropores materials can accelerate the migration rate of ions in electrolytes, improve multiplier performance and circle performance of the NPCs.

**Figure 6:**
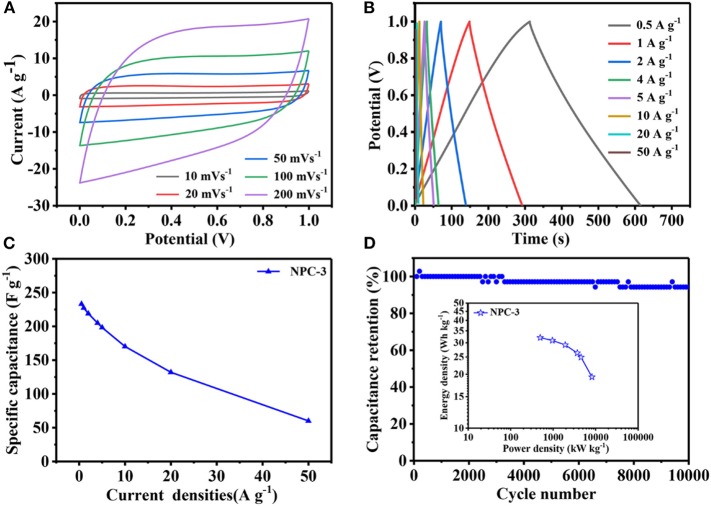
**(A)** Electrochemical performance of NPC-3 tested in a two-electrode system. **(B)** GCD curves of NPC-3 at various current densities. **(C)** Specific capacitance of NPC-3 under variable current densities. **(D)** Cycling performance of NPC-3 at 10 A/g, the inset is Ragone plots before 10,000 cycles.

The NPCs have been assembled into symmetric supercapacitor to investigate their electrochemical performance. Figure 6A shows the NPC-3 cyclic voltammetry curves, which is approximately rectangular, indicating that the material has good capacitance performance. When the scanning speed reaches 100 mV/s, the curve slightly changes but still maintains the shape of rectangle, indicating that NPC-3 has good capacitance retention. Because of the NPCs' unique hierarchical porous structure and high specific surface area, the GCD curve (Figure 6B) presents the shape of a nearly symmetrical triangle, and its current density ranges from 0.5 to 50 A/g, representing highly reversible charge-discharge behavior. The capacitance reaches 227.2 F/g at 1 A/g. In addition, there is still a specific capacitance of 170.0 F/g at 10 A/g with high capacitance retention (73.9%) (Figure 6C). As recorded in Figure 6D, the capacitance of NPC-3 has been reduced after 10,000 cycles at 10 A/g, but it also can maintain 94.2% of the initial specific capacitance. It is well-known that power density/energy density is an important parameter to evaluate the quality of supercapacitors (Tang et al., 2015). Due to the incorporation of N and P elements and the design of graded porous structure, the power/energy density of NPCs is up to 3694.084 W/kg and 26.289 Wh/kg, respectively.

## Conclusion

In this work, ED-derived porous carbon has been prepared through carbonization and activation at elevated temperature. The introducing of trace elements N and P influences the electron conductivity, ions adsorption and capacitive stability of the matrix, and endows the products with excellent electrochemical performance. Besides, the as-prepared samples show high specific surface area because of the abundant hierarchical porous structure. In three-electrode testing system, NPC-3 exhibits a high specific capacitance (340 F/g at 1 A/g) and excellent rate capacity (190 F/g at 100 A/g). Furthermore, in two-electrode configuration, the corresponding materials also maintains superb electrochemical performance (227.2 F/g at 1 A/g and 170.0 F/g at 10 A/g). Its high energy/powder density (26.289 Wh/kg at a power density of 3694.084 W/kg) and good cycling stability ensure NPC holds great application promise for high-performance supercapacitor.

## Data Availability Statement

All datasets generated for this study are included in the article/Supplementary Material.

## Author Contributions

JZ and SY were responsible for literature searching and drafting. All authors contributed equally to the final writing of the paper.

### Conflict of Interest

The authors declare that the research was conducted in the absence of any commercial or financial relationships that could be construed as a potential conflict of interest.
