# Supplementary material for: Nitrogen and Phosphorus Co-doped Porous Carbon for High-Performance Supercapacitors
Source: Front Chem. 2020 Feb 20;8:105. doi: 10.3389/fchem.2020.00105 (PMC7045068; doi:10.3389/fchem.2020.00105)
Supplement: Supplementary file 1 [file Data_Sheet_1.docx]

Supplementary Material

Nitrogen and phosphorus co-doped porous carbon for high-performance supercapacitors

**Jiaming Zhou^1^, Shewen Ye^1^, Qinqin Zeng^1^, Hui Yang****^1^, Jiahao Chen^1^, Ziting Guo^1^,** **Honghui Jiang^1,^*, Karthikeyan Rajan^1,^***

^1^School of Materials Science and Engineering, Jiangxi University of Science and Technology, Ganzhou, China

*** Correspondence:**Corresponding Author
jhonghui@163.com; karthikeyan148@gmail.com

**Electrochemical measurements**

For the three-electrode system, the specific capacitance of GCD was calculated according to the following equation (1):

$$\begin{aligned} \text{C}_{\text{s}}^{\text{*}}\text{=}\frac{\text{I×}\text{}\text{t}}{\text{m×}\text{}\text{V}}\#\left( \text{1} \right) \end{aligned}$$

For the two-electrode system, the specific capacitance of GCD was calculated according to the following equation (2):

$$\begin{aligned} \text{C}_{\text{s}}\text{=}\frac{\text{2×I×}\text{}\text{t}}{\text{m×}\text{}\text{V}}\#\left( \text{2} \right) \end{aligned}$$

Where I (A) is the discharge current, m (g) is the mass of the active materials in a single electrode and ΔV (V) is potential window. The energy density (E, Wh/kg) and power density (P, W/kg) was calculated according to the following equations:

$$\begin{aligned} \text{E}\text{=}\frac{\text{C}_{\text{s}}\text{×}{\text{}\text{V}}^{\text{2}}}{\text{2×}\text{3.6}}\#\left( \text{3} \right) \end{aligned}$$

$$\begin{aligned} \text{P}\text{=}\frac{\text{3600×}\text{E}}{\text{}\text{t}}\#\left( \text{4} \right) \end{aligned}$$

Where C_s_ (F/g) is the specific capacitance of a two electrode device, ΔV (V) is potential window and Δt is the discharge time (s).


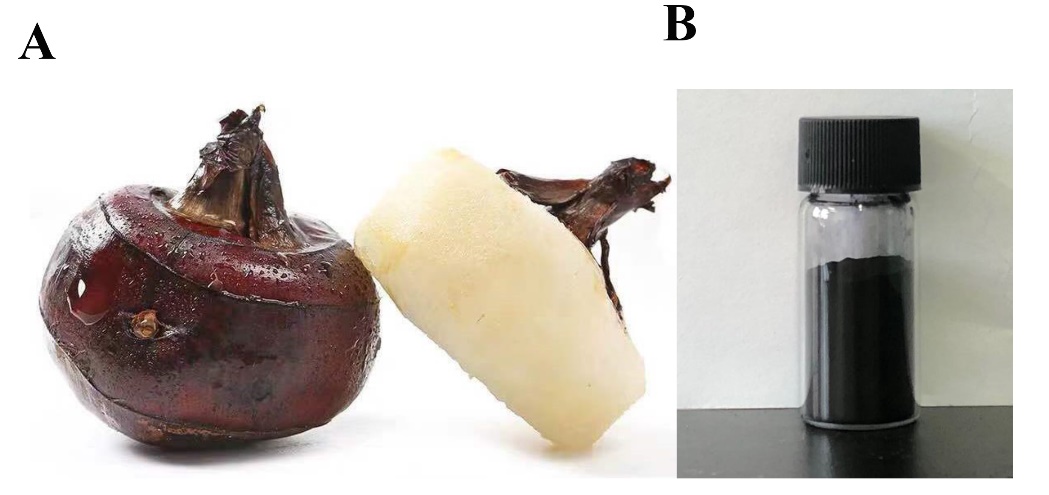


**FIGURE. S1** optical images of ED before (A) and after (B) carbonization


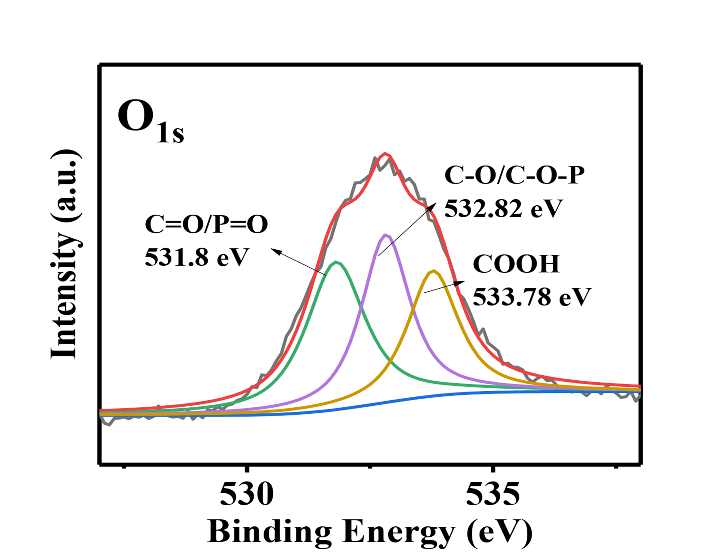

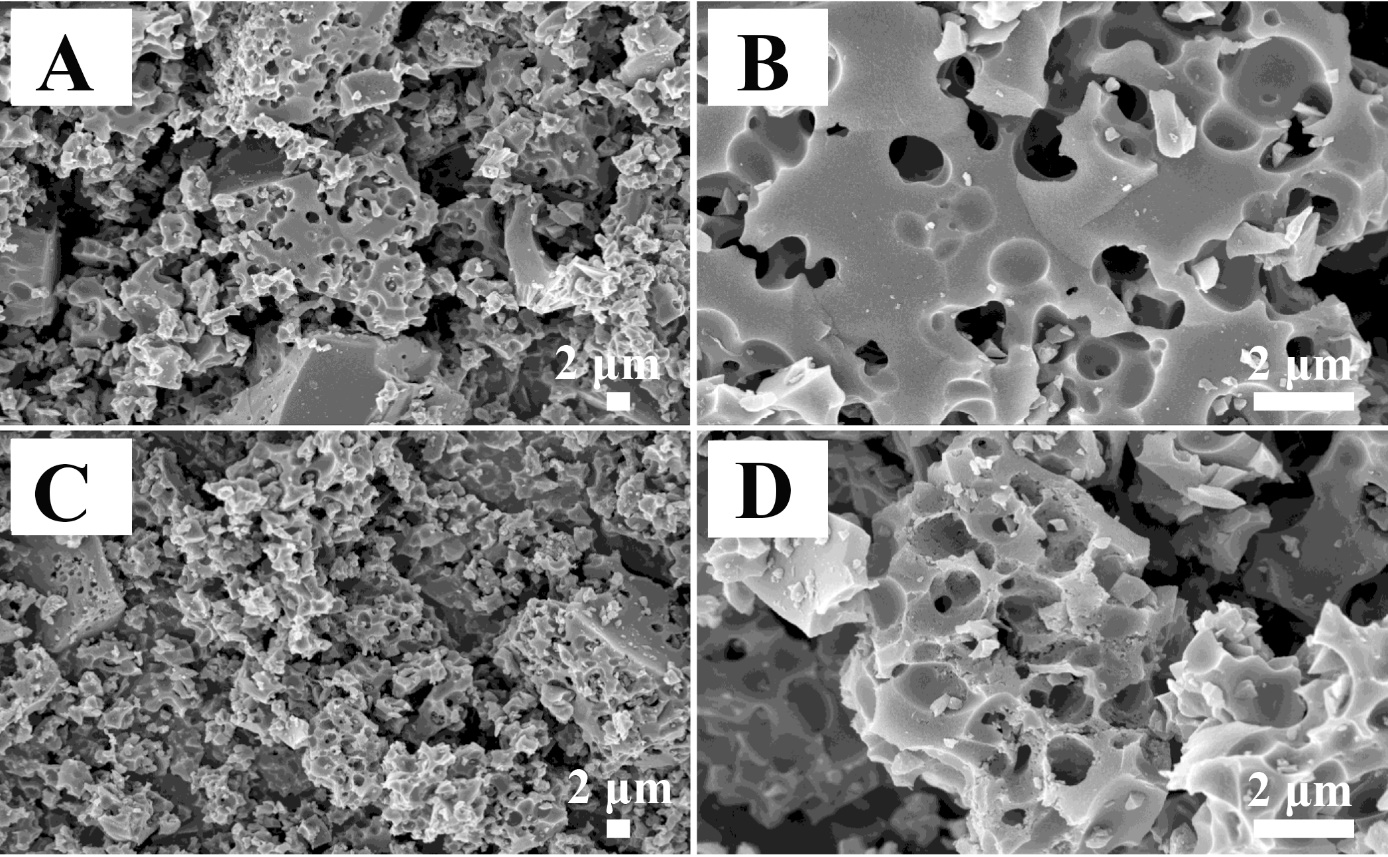
**FIGURE. S2** (A, B) SEM images of NPC-1. (C,D) SEM images of NPC-2

**FIGURE. S3** O_1s_ XPS spectra for NPC-3


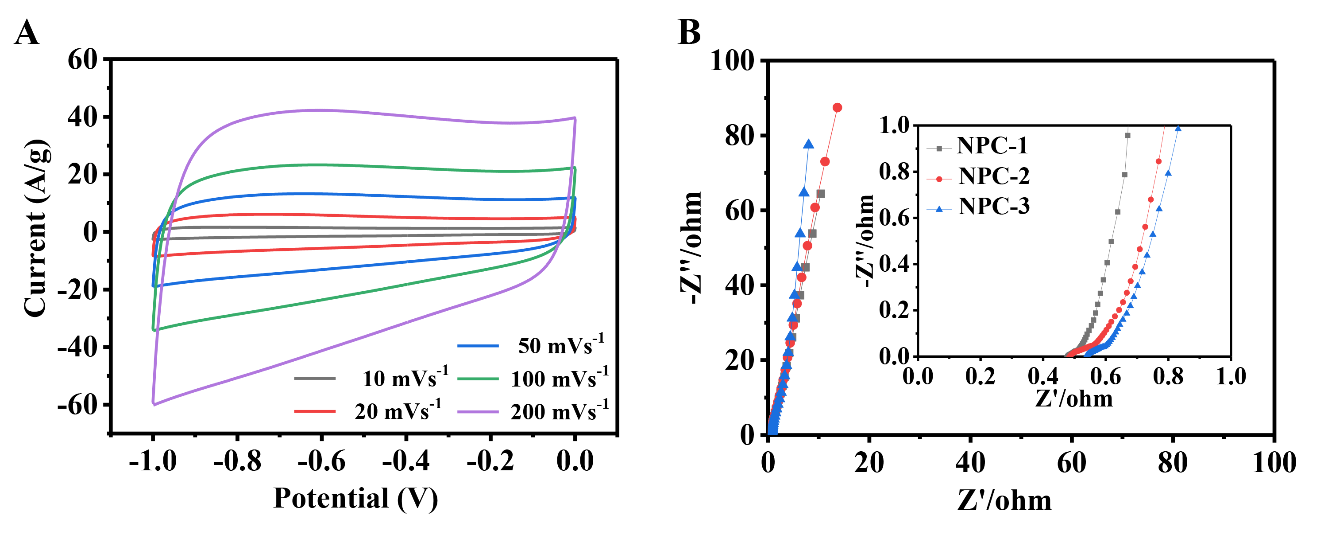


**FIGURE. S4** (A) CV curves of NPC-3 at different scanning rates; (B) Nyquist plot of NPCs

**Table.S1**. different types of N in PCs according to the XPS results

| Samples | Pyridine-N  (%) | Quaternary-N  (%) | Pyrrolic-N  (%) |
| --- | --- | --- | --- |
| NPC-1 | 36.5 | 32.1 | 31.3 |
| NPC-2 | 32.9 | 24.7 | 42.4 |
| NPC-3 | 37.2 | 17.4 | 45.4 |

**Table.S2**. Comparison of BET, relative atomic concentrations of the N and P and electrochemical performance of carbon materials derived from biomass precursors

| Biomass precursor | S _BET_  (m^2^ g^−1^) | N content  (at%) | P content  (at%) | Cs  (F g^−1^) | Measurement  condition | Ref |
| --- | --- | --- | --- | --- | --- | --- |
| Fish scale | 1134.20 | 5.74 | 32.1 | 332 | 6 M KOH  1 A/g | (Wang et al., 2015) |
| Elaeocarpus tectorius shell | 860.00 | 0 | 8.10 | 201 | 1 M H_2_SO_4_  1 A/g | (Nirosha et al., 2020) |
| Silkworm Cocoon | 1247.60 | 3.56 | 1.63 | 317 | 1 M H_2_SO_4_  1 A/g | (Wang et al., 2019) |
| Shrimp shell | 725.60 | 3.34 | 1.77 | 206 | 6 M KOH  0.1 A/g | (Qu et al., 2015) |
| Shiitake | 2335.00 | 1.10 | 1.60 | 283 | 6 M KOH  0.5 A/g | (Cao et al., 2016) |
| Silkworm excrement | 2258.00 | 2.15 | 0.26 | 401 | 6 M KOH  0.5 A/g | (Lei et al., 2018) |
| Eleocharis dulcis | 2454.00 | 0.95 | 0.18 | 340 | 6 M KOH  1 A/g | This work |

**References**

Cao, H., Chen, Z., Chen, Q., Yang, C., Hou, L., Rehan, M., et al. (2016). A shiitake-derived nitrogen/oxygen/phosphorus co-doped carbon framework with hierarchical tri-modal porosity for high-performance electrochemical capacitors. *RSC Advances* 6(85)**,** 81527-81533. doi: 10.1039/c6ra13689c.

Lei, S., Chen, L., Zhou, W., Deng, P., Liu, Y., Fei, L., et al. (2018). Tetra-heteroatom self-doped carbon nanosheets derived from silkworm excrement for high-performance supercapacitors. *J. Power Sources* 379**,** 74-83. doi: 10.1016/j.jpowsour.2018.01.032.

Nirosha, B., Selvakumar, R., Jeyanthi, J., and Vairam, S. (2020). Elaeocarpus tectorius derived phosphorus-doped carbon as an electrode material for an asymmetric supercapacitor. *New J. Chem.* 44(1)**,** 181-193. doi: 10.1039/c9nj04813h.

Qu, J., Geng, C., Lv, S., Shao, G., Ma, S., and Wu, M. (2015). Nitrogen, oxygen and phosphorus decorated porous carbons derived from shrimp shells for supercapacitors. *Electrochim. Acta* 176**,** 982-988. doi: 10.1016/j.electacta.2015.07.094.

Wang, J., Shen, L., Xu, Y., Dou, H., and Zhang, X. (2015). Lamellar-structured biomass-derived phosphorus- and nitrogen-co-doped porous carbon for high-performance supercapacitors. *New J. Chem.* 39(12)**,** 9497-9503. doi: 10.1039/c5nj02080h.

Wang, Y., Zhang, M., Dai, Y., Wang, H.-Q., Zhang, H., Wang, Q., et al. (2019). Nitrogen and phosphorus co-doped silkworm-cocoon-based self-activated porous carbon for high performance supercapacitors. *J. Power Sources* 438. doi: 10.1016/j.jpowsour.2019.227045.
